# High‐Throughput Screening of Blade‐Coated Polymer:Polymer Solar Cells: Solvent Determines Achievable Performance

**DOI:** 10.1002/cssc.202101888

**Published:** 2022-01-21

**Authors:** Albert Harillo‐Baños, Qunping Fan, Sergi Riera‐Galindo, Ergang Wang, Olle Inganäs, Mariano Campoy‐Quiles

**Affiliations:** ^1^ Institut de Ciència de Materials de Barcelona (ICMAB-CSIC) Carrer dels Til⋅lers s/n Campus UAB Bellaterra 08193 Spain; ^2^ Department of Chemistry and Chemical Engineering Chalmers University of Technology Göteborg 412 96 Sweden; ^3^ Biomolecular and Organic Electronics Department of Physics, Chemistry and Biology (IFM) Linköping University Linköping 581 83 Sweden

**Keywords:** energy conversion, Hansen solubility parameters, high-throughput screening, organic photovoltaics, solar cells

## Abstract

Optimization of a new system for organic solar cells is a multiparametric analysis problem that requires substantial efforts in terms of time and resources. The strong microstructure‐dependent performance of polymer:polymer cells makes them particularly difficult to optimize, or to translate previous knowledge from spin coating into more scalable techniques. In this work, the photovoltaic performance of blade‐coated devices was studied based on the promising polymer:polymer system PBDB‐T and PF5‐Y5 as donor and acceptor, respectively. Using the recently developed high‐throughput methodology, the system was optimized for multiple variables, including solvent system, active layer composition, ratio, and thickness, among others, by fabricating more than 500 devices with less than 24 mg of each component. As a result, the power conversion efficiency of the blade‐coated devices varied from 0.08 to 6.43 % in the best device. The performed statistical analysis of the large experimental data obtained showed that solvent selection had the major impact on the final device performance due to its influence on the active layer microstructure. As a conclusion, the use of the plot of the device efficiency in the Hansen space was proposed as a powerful tool to guide solvent selection in organic photovoltaics.

## Graphical abstract

**High‐throughput screening**: Less than 25 mg of the donor and acceptor materials are used to make over 648 devices in a combinatorial screening method that investigates processing parameters for blade‐coated polymer:polymer photovoltaics. Representing power conversion efficiency in the Hansen solubility space may be the key for easily selecting solvent systems that optimize performance.

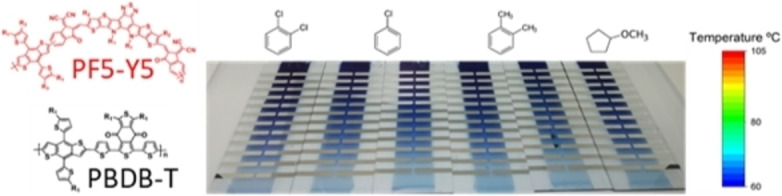

## Introduction

In recent years, solution‐processed organic photovoltaic (OPV) performance has improved significantly, surpassing power conversion efficiencies (PCEs) of 18 %.[^1^, ^2^] All‐polymer solar cells (all‐PSCs) have attracted increasing attention in the OPV field, as they exhibit several advantages with respect to polymer donor:small molecular acceptor solar cells, such as better thermal and mechanical stability, as well as improved processing versatility for up‐scaling.[^3^, ^4^, ^5^, ^6^] All‐PSCs currently exhibit, however, overall slightly lower PCEs than their polymer:small molecule counterparts, although the gap is rapidly diminishing.[^5^, ^7^, ^8^] For many combinations, the lower performance is mainly due to the formation of non‐optimal film morphologies, and in some cases, lower absorption coefficients than state‐of‐the‐art molecular acceptors, such as O‐IDTBR ((5Z,5′Z)‐5,5′‐((7,7′‐(4,4,9,9‐tetraoctyl‐4,9‐dihydro‐s‐indaceno[1,2‐b:5,6‐b′]dithiophene‐2,7‐diyl)bis(benzo[c][1,2,5]thiadiazole‐7,4‐diyl))bis(methanylylidene))bis(3‐ethyl‐2‐thioxothiazolidin‐4‐one)) or ITIC (3,9‐bis(2‐methylene‐(3‐(1,1‐dicyanomethylene)‐indanone))‐5,5,11,11‐tetrakis(4‐hexylphenyl)‐dithieno[2,3‐d:2’,3’‐d’]‐s‐indaceno[1,2‐b:5,6‐b’]dithiophene),^[9]^ and to lower charge carrier mobilities (electrons especially).[^7^, ^10^]

**Figure 1:**
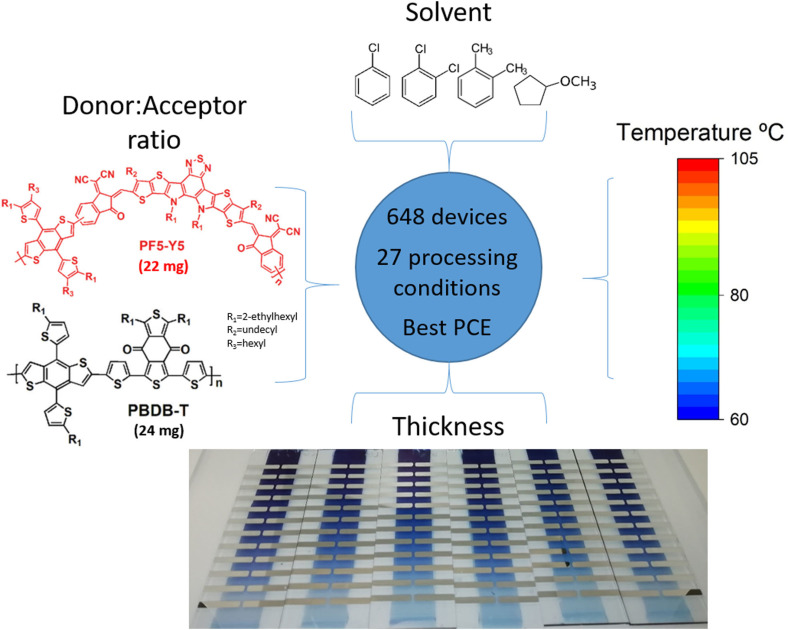
Schematic of the processing conditions for high‐throughput screening of polymer:polymer solar cells.

The lower electron mobility issue has been addressed by designing polymer acceptors based on naphthalene diimide (NDI) like P(NDI2OD‐T2) (commercially known as N2200)(Poly{[N,N′‐bis(2‐octyldodecyl)naphthalene‐1,4,5,8‐bis(dicarboximide)‐2,6‐diyl]‐alt‐5,5′‐(2,2′‐bithiophene)),[^7^, ^10^, ^11^] or perylene diimide (PDI),[^12^, ^13^] and lately on novel non‐fullerene acceptor (NFA) molecules,[^8^, ^14^, ^15^] like the polymer used in this work, PF5‐Y5 (see Figure 1 for the structure).^[16]^ The electron mobility of the latter has been reported to be 3.18×10^−3^ cm^2^ V^−1^ s^−1^, very close to the mobility of Y5 small‐molecule acceptor (3.82×10^−3^ cm^2^ V^−1^ s^−1^), which acts as the electron‐deficient unit for PF5‐Y5, and higher than ITIC (3.58×10^−4^ cm^2^ V^−1^ s^−1^), one of the more widely used NFAs.^[17]^ Moreover, PF5‐Y5 displays an improved absorption coefficient compared to its small‐molecule acceptor counterpart Y5.^[16]^

On the other hand, the film morphology issue is very intrinsic of polymer mixtures as it arises from the low entropy of mixing found in large‐molecular‐weight systems. Following Flory–Huggins theory, the very limited degrees of freedom accessible to most atoms in the covalently bonded polymer make the entropy of mixing small, and thus the tendency to demix pronounced.[^18^, ^19^] A stronger π‐orbital overlap at the donor/acceptor (D/A) interfaces is formed when the polymer donor and the polymer acceptor are aligned face‐to‐face, thereby reducing the exciton binding energy and promoting the formation of free charge carriers in all‐PSCs.[^20^, ^21^] Many efforts have been addressed to improve the morphology of all‐PSCs by modifying the polymer structure, like side‐chain engineering of the polymer backbone,[^22^, ^23^] developing random copolymers,[^24^, ^25^] or optimizing the molecular weight of the polymers.^[26]^ Moreover, from a practical point of view, there are many examples of controlling the film morphology of all‐PSCs blends by optimizing the deposition parameters; some examples include the use of different additives and solvents,[^16^, ^17^, ^27^, ^28^] thermal and solvent annealing,[^29^, ^30^] sequentially depositing the polymers (layer‐by‐layer),[^31^, ^32^, ^33^] mixed‐flow microfluidic,^[34]^ and even ternary systems.[^4^, ^5^, ^35^]

In most cases, spin coating with rapidly evaporating solvents is used to freeze the solution microstructure and thus prevent large‐scale phase separation. With polymer:polymer blends in solution, the higher viscosity due to polymers will restrict diffusion and tend to retain the solution glassy‐like blend structure. This approach cannot be readily transferred to other more scalable methods, such as blade coating, where slower rates of evaporation are found, and the drying period is often extended over many seconds. Also, the parameter space for optimization is very large, and large amounts of material are required to scan the different processing avenues. Besides, there may be fundamental limitations regarding how fast the solvent can be removed in a process such as blade coating, compared to spin coating. This, together with the lack of rationales for morphology control, has prevented all‐PSCs from moving beyond lab scale. We note, however, the recent appearance of highly efficient all‐PSCs over 1 cm^2^ areas, albeit depositing the active layer again by spin coating.^[36]^

In this work, we investigate the importance of different deposition parameters, including solvent selection and solubility, in the final efficiency of devices based on PBDB‐T:PF5‐Y5 D/A blends. The corresponding chemical structures are shown in Figure 1. This system has resulted in one of the highest polymer:polymer efficiencies thus far, reaching 14 % when optimized and processed by spin coating.^[16]^ We have employed our high‐throughput methodology recently developed to screen blade‐coating conditions for all‐PSCs.^[37]^ Similar high‐throughput methodology has been also reported recently to optimize the composition of ternary blends.^[38]^ Using only 22 mg of D and 24 mg of A, we have produced more than 500 devices and scanned 27 different processing conditions (Figure 1). Statistical analysis based on ANOVA^[39]^ shows that the parameters related to the solvent characteristics (boiling point and Hansen solubility parameters) are the most important factors correlating to efficiency. Additional spectroscopic measurements on the devices provide further insights into the morphology and its correlation with performance. Moreover, we propose to plot efficiency in the Hansen space (without the need to actually measure the Hansen parameters of the materials) as a tool to find appropriate solvents, that is, solvents that produce microstructures that optimize device performance.

## Results and Discussion

The corresponding chemical structures are shown in Figure 1. PF5‐Y5 was synthesized following previous reports.^[16]^ The polymers present complementary absorption spectra and their energy levels allow to obtain high open‐circuit voltage (see Figure S1 in the Supporting Information). In total, we have fabricated 648 devices by blade coating, with 26 different processing conditions, including 10 solvent systems, 6 D/A composition ratios (1 : 0.5, 1 : 0.75, 1 : 0.9, 1 : 1, 0.75 : 1, 0.5 : 1, 0.25 : 1, 0.11 : 1), 3 deposition temperatures (60, 80, and 105 °C), and sequential depositions. Additionally, all samples were fabricated with active layer thickness gradients by means of variable speed blade during deposition, in order to deepen the exploration of the parameter space at minimum material cost.^[40]^ These thickness gradients were fabricated by linearly decelerating the blade during deposition from 90 to 10 mm s^−1^, within the 7.5 cm length of substrate (each divided in 24 devices). Higher speeds produce thicker films, so by decelerating we obtain a gradient from a thicker to a thinner layer because we are working in viscous drag regime (also called Landau–Levich regime). Moreover, the thickness also depends on the amount of solution left in front of the blade, becoming thinner as the solution runs out; we minimized the thickness differences from batch to batch by depositing the same amount of solution in each deposition. The combination of these two effects increases the gradient slope. Typically we have obtained thicknesses from 100 to 30 nm in a single substrate (see Figure S2).^[37]^ The entire study was done using only 22 mg of PBDB‐T and 24 mg of PF5‐Y5, averaging to 0.07 mg of total material per pixel, where each pixel has an area of 8 mm^2^. This 0.07 mg average takes into account all the losses of the manufacturing, like excess solution and removed or non‐contacted active layer. For comparison purposes, an equivalent study but without high‐throughput methodology, which used spin coating, would have required around 1.2 mg per data point, or a total of 330 mg of each material.^[41]^ The optimal composition ratio was found to be 1 : 0.75 D/A, but, as mentioned, more ratios were explored for completeness.^[16]^

**Figure 2:**
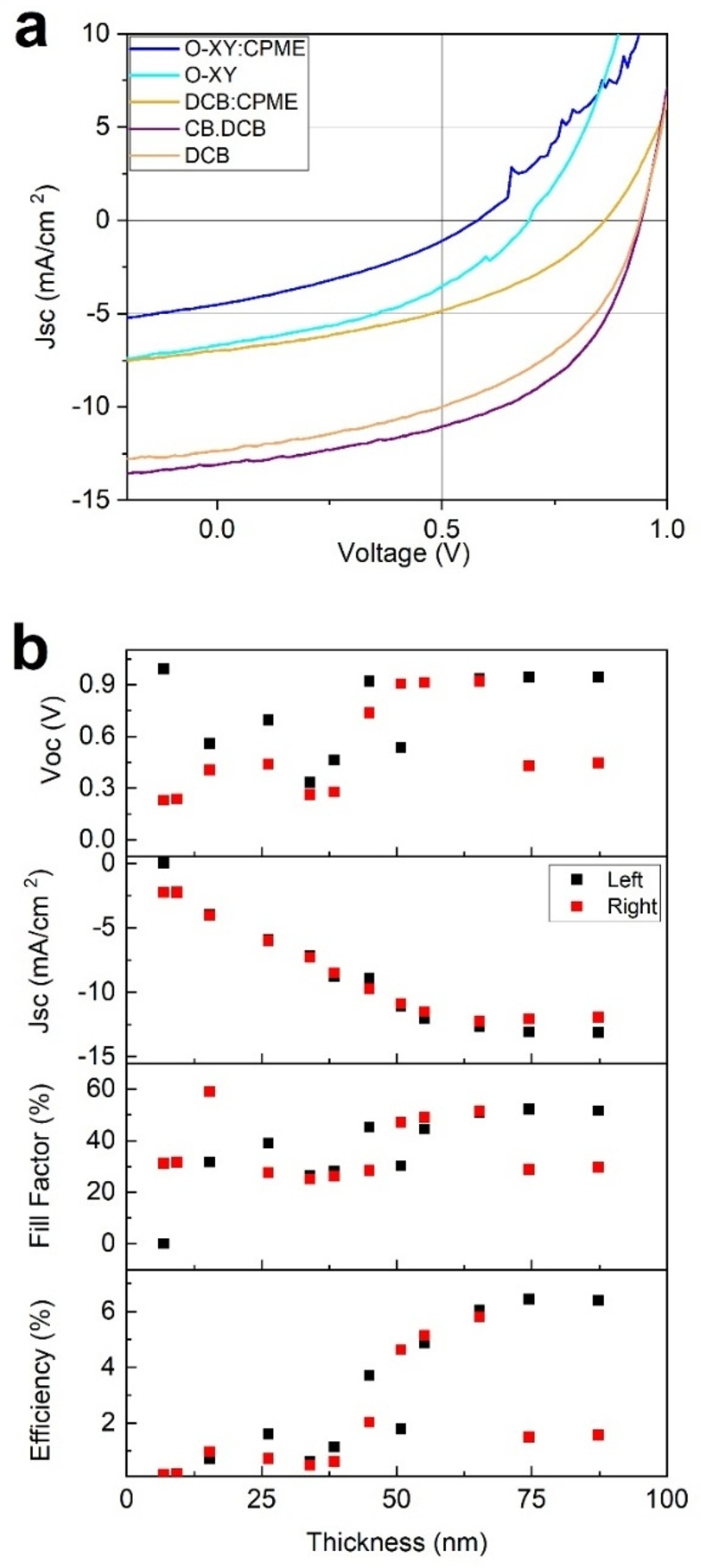
(a) *J*–*V* curves for one of the best cells for each of the five best solvent systems (O‐XY, DCB, CB:DCB, CPME/O‐XY, and DCB/CPME) (b) *J*_sc_, *V*_oc_, FF, and PCE as a function of active layer thickness for each pixel for one of the best solvents (CB/DCB). Each substrate contains 24 pixels, 12 on either side (labelled left and right). The thickness gradient is expected to be along the long axis of the substrate, leaving two nominal duplicates per thickness.

The solvents used for this study were some of the most widely used solvents in OPV processing, namely *o*‐xylene (O‐XY), chlorobenzene (CB), and dichlorobenzene (DCB), as well as cyclopentyl methyl ether (CPME), a promising green solvent.[^42^, ^43^] Some relevant properties of these solvents, such as boiling point and Hansen parameters,^[44]^ are displayed in Table 1. Devices were fabricated with active layers that use these four solvents and binary mixtures thereof, usually by mixing a 1 : 1 volume ratio. Figure 2a shows representative current density–voltage (*J*–*V*) curves for different solvents, and Figure 2b gives an example of the use of thickness gradients to determine optimum thickness for the solvent system, which leads to a higher performance. Additionally, thickness‐dependent data for other systems can be found in the Supporting Information (Figures S2, S4–S8). Even though in organic solar cells the optimized active layer thickness is generally over 100 nm, using blade coating of PBDB‐T and PF5‐Y5, the optimum thickness is below 100 nm. Probably, this may be because the microstructure of the polymers limits the charge transport, resulting in optimum efficiency for thinner active layers.^[45]^

**Table 1:** Solvent properties.

Short name	Boiling point [°C]	δ*D*	δ*P*	δ*H*	Structure
CPME	106	16.7	4.3	4.3	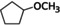
O‐XY	144	17.8	1.0	3.1	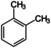
CB	132	19	4.3	2.0	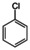
DCB	180	19	6.3	3.3	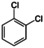

Table 2 shows the D/A ratio, deposition temperature, and blade speed that led to highest performance for each solvent system, as well as the number of devices and the analyzed processing conditions (Notes). The final PCEs of the devices vary greatly depending on the deposition parameters with PCE values ranging from 0.08 to 6.4 %. Moreover, the photovoltaic parameters obtained for the same solvent system are different when comparing spin coating and blade coating, as the two techniques can lead to different film morphologies due to their different drying mechanisms and times.^[46]^ The devices with higher performance were those that used DCB as part of the solvent system. In Figure S10, the external quantum efficiencies (EQEs) of PBDB‐T:PF5‐Y5 at 1 : 1, 1 : 4, and 1 : 8 D/A ratio using DCB are shown. Devices with D/A ratio 1 : 1 have better performance, showing a deep valley at 700 nm in the EQE for thinner active layers; this effect decreases as the thickness of the active layer increases. Interestingly, at D/A ratio 1 : 4, devices with thicker active layer show higher EQE in the donor region than the acceptor region. Finally, devices with D/A ratio 1 : 8 have a poor performance, showing an EQE peak at 800 nm. On the other hand, employing CB as the only solvent resulted in very low PCE values. Interestingly, when using the mixed CB/DCB system, we obtained the highest PCE. Similarly, the devices were significantly improved when using DCB/CPME instead of just CPME. This might lead to the conclusion that, in solvent mixtures, the solvent with highest boiling point lasts longer during the deposition and, thus, is the one that has the greatest impact on the final morphology and the concomitant PSC performance. This is a similar mechanism to what is observed in some cases when additives are used.^[47]^

**Table 2:** Conditions that led to the highest efficiency for each solvent, and corresponding device data.^[a]^

Solvent system	D/A	*T* [°C]	Blade speed [mm s^−1^]	*V*_oc_ [V]	*J*_sc_ [mA cm^−2^]	FF [%]	PCE [%]	Number of devices per solvent system	Notes
CPME	1 : 0.9	60, 80, 105	90	0.77	−1.25	37.1	0.36	72	thickness, *T*
CPME/O‐XY (0.65 : 0.35)	0.75 : 1	80	83	0.69	−4.20	33.6	0.97	48	thickness
CPME/O‐XY (1 : 1)	1 : 0.5	80	90	0.77	−4.24	48.4	1.57	120	thickness, D/A ratio
O‐XY	1 : 0.9	80	90	0.75	−7.61	35.8	3.17	144	thickness, D/A ratio
O‐XY	1 : 0.9	80	61	0.91	−10.76	53.0	5.20	24	thickness, 2 layers^[b]^
CB	1 : 0.5	80	25	0.44	−2.33	28.5	0.30	24	thickness
DCB	1 : 0.75	105	61	0.94	−12.41	51.8	6.02	96	thickness, D/A ratio
CB/DCB (1 : 1)	1 : 1	105	83	0.94	−13.09	52.1	6.43	24	thickness
DCB/O‐XY (1 : 1)	0.5 : 1	105	32	0.88	−8.26	49.3	3.59	48	thickness, D/A ratio
CB/O‐XY (1 : 1)	1 : 0.75	80	10	0.38	−0.67	30.7	0.08	24	thickness
DCB/CPME (1 : 1)	1 : 0.75	105	54	0.57	−20.02	35.7	4.04	24	thickness

[a] *V*_oc_: open‐circuit voltage; *J*_sc_: short‐circuit current density; FF: fill factor. Notes indicates the screened fabrication parameters [b] The active layer was deposited twice in a sequential deposition, keeping the thickness gradient in both layers.

Other factors, such as pre‐aggregation of the polymers in solution, will strongly depend on the solvent and solvent mixture and could have strong effects on the final microstructure and concomitant photovoltaic performance.^[48]^ From the above discussion, it seems clear that predicting which deposition conditions (including choice of solvent) are required for obtaining high efficiencies is not straightforward.

To determine which of the deposition parameters affects more strongly the final PCE, we performed a One‐Way ANOVA analysis for several parameters of all of the fabricated devices.[^49^, ^50^] This analysis results in a parameter, namely F factor, which quantifies how much the target magnitude (PCE in this case) varies when the given parameter is scanned. In other words, it is a statistical measure of the importance of a given parameter in the final efficiency. We analyzed the importance of the D/A ratio, the used blade speed for each pixel (as a proxy for active layer thickness), the boiling point, and Hansen solubility parameters to characterize each solvent. Deposition temperature was not analyzed in detail as different temperatures for the same system (CPME) resulted in negligible changes in performance. Results are shown in Table 3, where, as mentioned, larger F factors mean larger impact in the final PCE. The ANOVA analysis indicates that, within the relatively large parameter space explored, solvent selection is the parameter that has the largest impact on final PCE of the devices, even compared to thickness of the active layer and D/A composition ratio. A note of caution should be given here, as the ANOVA analysis only considers the existing data, and we have not fabricated cells with extreme thickness (e. g., μm) or composition values (e. g., 1 : 0), but rather typical (but relatively wide) range of values that result in efficient cells as described in the experimental section and in Figures 2b and S3–S7.

**Table 3:** ANOVA analysis of the parameters varied during the solar cell fabrication.

Property	F
blade speed	6
D/A ratio	14
δ*D*	45
δ*P*	68
δ*H*	46
boiling point^[a]^	46
boiling point^[b]^	110

[a] For mixtures, average value between solvents. [b] For mixtures, value of the highest boiling point amongst components.

**Figure 3:**
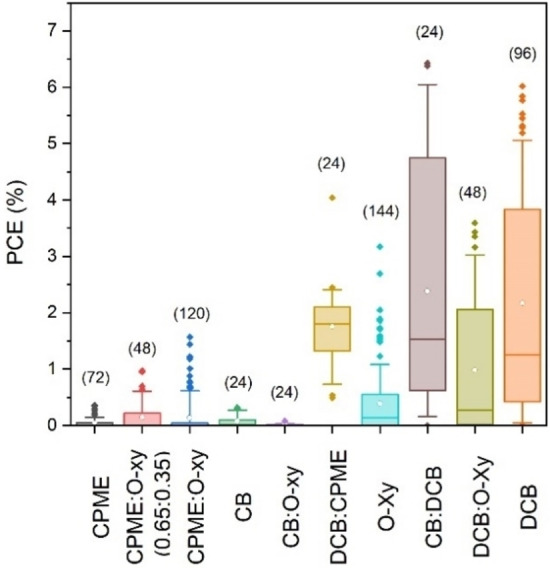
Statistical representation of all samples fabricated as a function of solvent (with increasing solvent boiling point). In parentheses is the number of devices fabricated per solvent. Each box contains 25th to 75th percentile of all the PCE for a solvent or mixture, while whiskers cover 10th to 90th. White dots inside the boxes are the mean PCE of the solvent, and colored dots outside the boxes are outliers above 90th or below 10th percentile.

As solvent appears to be the most important of the explored parameters, we represented in a box plot the PCE of all the devices fabricated for each different solvent and mixture. In Figure 3, the solvent systems used in this work are ordered from lower to higher boiling point on the *x*‐axis. The observed trend between higher boiling point and higher PCE could be related with the slower evaporation rates in blade coating depositions, which would allow a morphological reorganization of the polymer:polymer system.

**Figure 4:**
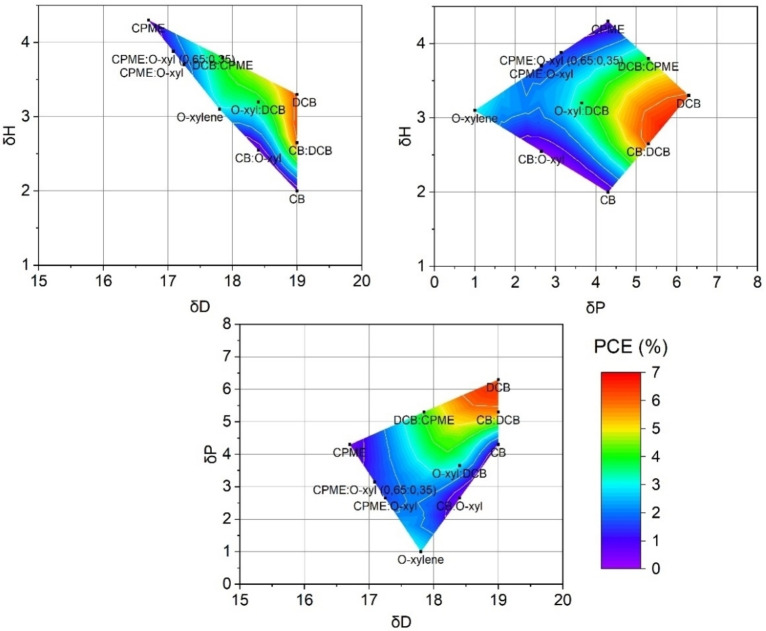
Performance landscape of polymer:polymer solar cells represented on the Hansen solubility space.

In light of the results of the ANOVA analysis (see Table 3), we decided to analyze the role of the solubility by plotting the PCE in the Hansen parameter space, assigning to each PCE value the Hansen coordinates of the corresponding solvent used for the preparation of the active layer of the device. The corresponding 2D projections of the 3D Hansen space are plotted in Figure 4.

In this representation, we obtain a continuous landscape with two PCE maxima and a deep valley in between. This double maximum in PCE might be due to different reasons, such as a difference in solubility of the two materials, the interaction between solvents, or the possibility of specific solvents promoting aggregation in solution. It is noteworthy to mention than we did not calculate the Hansen solubility parameters of both materials, and therefore we did not need the large quantities of materials that this kind of study requires. Instead, we find clear tendencies when plotting directly PCE. Also, in this representation, the maximum does not represent the maximum solubility, but rather the optimum solubility of the blend for solar cells, which has a more direct application in device fabrication and optimization. We would like to explain that our original data set included 80 % of the data points currently shown in Figure 4. After seen the initial version of the plotted data, we decided to make an initial evaluation of the predicting capability of the Hansen efficiency surface by fabricating cells using two additional solvent mixtures. We prepared CB/O‐XY‐ and DCB/CPME‐based devices, included in Figure 4, and found that they fitted into the surface. While a full evaluation of the predicting capability of this approach would require a larger subset of solvents and solvent mixtures, these results strongly suggest that this representation can be useful to identify good solvents in all‐PSCs.

**Figure 5:**
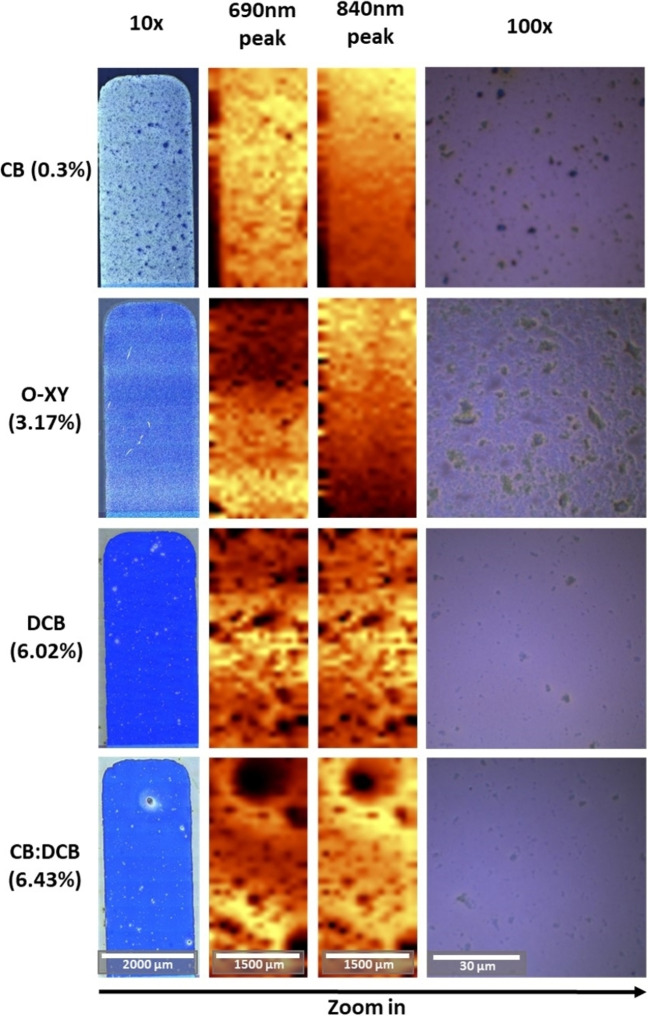
For the best device fabricated with each solvent (PCE in parentheses), from left to right: microscope image of device with 10× objective, integration of the 690 and 840 nm PL peaks, and microscope image with 100× objective, respectively. Note: PL measurements do *not* share the same intensity (color) scale.

In order to understand the differences in performance of the devices fabricated based on different solvents, we evaluated the same device samples using a variety of microscopic and spectroscopic tools. Since our target was to perform the study minimizing the raw material employed, we performed this study on full devices, only using films of the pristine materials as reference. Figure 5 shows obtained optical microscopy and photoluminescence (PL) images taken on pixels with highest performance for five of the solvents used. The PL images show position‐dependent integrated intensity for two PL bands, one centered at 690 nm and the other one at 840 nm, corresponding to PL from the donor and acceptor materials, respectively (see Figure S3).

Samples fabricated from CB are inhomogeneous at all length scales. The 100× optical microscopy images (Figure 5) show that large crystals have formed in all devices, but they reduce in size and number with increasing PCE, as well as the layer being more homogeneous (i. e., DCB and CB/DCB samples). Despite some larger defects and crystals appearing in the best‐performing cells (10× images), the rest of the active layer is rather homogeneous, while in the less‐efficient devices the crystals are more omnipresent throughout the device. The integrated PL over the whole pixel scales with the film thickness and overall composition, and it is shown in Figure S3. Comparing the PL magnitude, there are thickness or composition variations at the pixel level for all the substrates (note that the PL images do not share Y scale).

To gain further insights into the differences between solvents, and inspired by the fact that different solvents resulted in different “optimum” thickness, we looked at the thickness dependence of the morphology. Figures S4–S9 show a set of images for pixels with different active layer thickness as a function of deposition solvent. One general observation is that the number of micron‐sized crystals strongly depend on thickness: thinner layers exhibit less crystallites. This could be due to the effect of geometrical confinement.^[51]^ Alternatively, it may be a result of pre‐aggregation: thicker films would naturally deposit a larger amount of solid content on the film, thus increasing the number of pre‐aggregated crystallites. The low device PCE of some of the solvents might be explained through this excessive crystallization and phase separation. Moreover, this also explains why the optimum thickness for the worse solvents is thinner than for good solvents (Figure S2).

**Figure 6:**
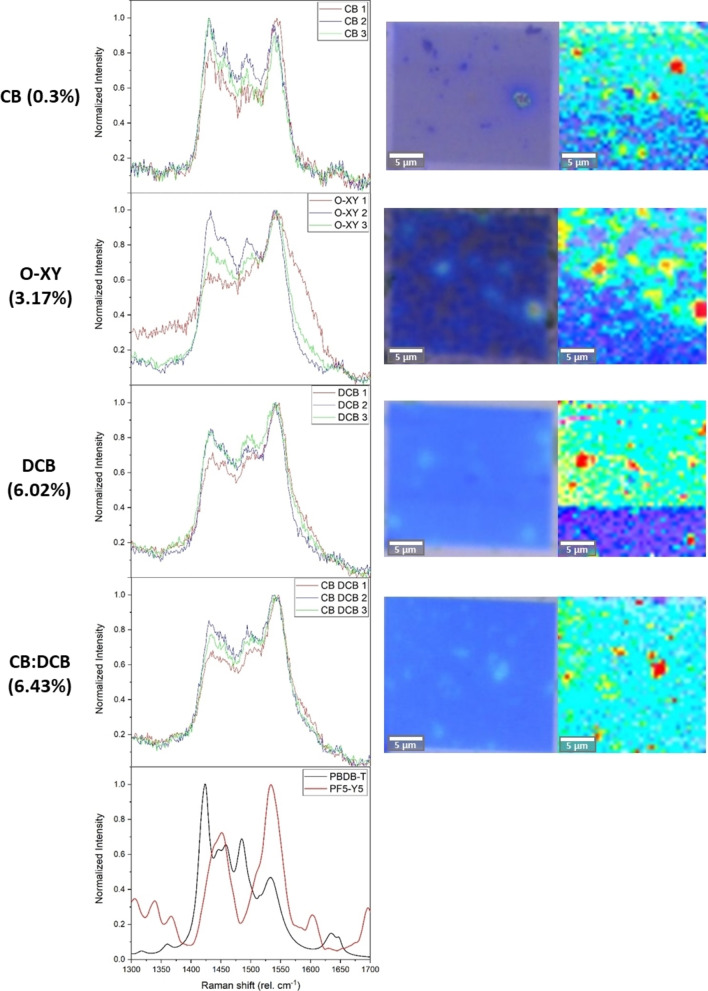
For the best device fabricated with each solvent (PCE in parentheses), left to right: comparison of the different spectra obtained in high‐resolution Raman imaging; 100× microscopy image of the mapped zone; Raman mapping of the components, with acceptor‐rich areas in red. Last plot is the reference Raman spectra of pure donor and pure acceptor. Note that, for clarity, the images do *not* share the same scale.

To understand the formation of these large aggregates, we performed high‐resolution Raman spectroscopy imaging in the same samples. This helped us to confirm the composition of the crystallites and the blend around them. Upon subtraction of the background and cosmic rays, the Raman data was investigated by principal components analysis and compared to the Raman spectra of each material. This analysis allows to evaluate differences in the local composition of D/A. The results of this analysis are summarized in Figure 6.

At this length scale (25 μm×25 μm) several morphological issues could be detected. The Raman analysis shows that there are indeed variations in the local composition, specifically at defects and/or crystallites, that are in most cases, very rich in acceptor. This is consistent with the stronger tendency to crystallize for PF5‐Y5 compared to the donor polymer, as reported in the literature by means of GIWAXS (Grazing‐Incidence Wide‐Angle X‐ray Scattering).^[16]^ If we take as proxy for the degree of phase separation the spectral difference between different regions, we can observe that the general trend is that films become more homogeneous when using solvents that lead to higher device efficiencies, probably due to unfavorable phase separation and/or crystallization of PF5‐Y5. We note, however, that the spatial resolution of this experiment is still low compared to the exciton diffusion length. Interestingly, while some solvents resulting in moderate (O‐XY) device efficiencies also exhibit variability in the blend composition at micron scale, as probed by Raman spectroscopy, the case of CB is different, as it looks particularly homogeneous in terms of composition judging by the similarity between spectra at different locations.

## Conclusions

We have demonstrated that one of the most important factors in the final power conversion efficiency (PCE) of all‐polymer solar cells (all‐PSCs) fabricated by blade coating is indeed solvent selection. We first found that there appears to be a minimum boiling point for the microstructure of the active layer obtained by blade coating to be acceptable. This is a necessary but not sufficient requirement as particular solubility values are also required. The importance of the solvent selection is exemplified with the appearance of large, micron‐sized aggregates of the acceptor (PF5‐Y5), which are either present in the solution or generated while depositing the layer and are detrimental to the performance of the device. The size and number of these crystallites depend on the thickness of the active layer and the solvent used, with more crystallites protruding in thicker layers. A consequence of this is that bad solvents only lead to functional devices for thin films, much thinner than the thickness the leads to the maximum interference of the electric field within the active layer. On the other hand, devices fabricated with better solvents can be fabricated thicker, yielding overall higher PCE.

The current study was done using our high‐throughput method, which allowed us to complete the study with less than 24 mg of each material. This is only a small fraction of the material that would have been required for the same study and the same number of samples (648 devices) following conventional methodologies. The number of devices is enough to apply simple statistical analysis of the relevance of the different parameters, from which solvent solubility and boiling point are highlighted. We propose the visualization of the PCE in the Hansen space as a tool to guide the selection of solvents in all‐PSC. Our methodology would allow to implement algorithms to find the most relevant processing parameter for optimal blend morphology in the complex structure–property relationships of all‐PSC, instead of the daunting trial‐and‐error approach, and consequently optimize the performance of all‐PSC.

## Experimental Section

### Materials

The glass substrates with patterned indium tin oxide (ITO; 100 nm thick) were purchased in Ossila. ZnO ink formulation was purchased from Avantama. PBDB‐T (Poly[(2,6‐(4,8‐bis(5‐(2‐ethylhexyl)thiophen‐2‐yl)‐benzo[1,2‐b:4,5‐b’]dithiophene))‐alt‐(5,5‐(1’,3’‐di‐2‐thienyl‐5’,7’‐bis(2‐ethylhexyl)benzo[1’,2’‐c:4’,5’‐c’]dithiophene‐4,8‐dione)]) was purchased from Brilliant Matters. PF5‐Y5 was synthesized as reported elsewhere.^[16]^ The polymers were dissolved in a concentration range of 15 mg mL^−1^. Molybdenum oxide (MoOx) was acquired from Alfa Aesar.

### Sample preparation

The substrates were cleaned by consecutive sonication baths in acetone, Hellmanex 10 vol % solution in water, isopropanol (5 min each), and sodium hydroxide 10 vol % (10 min), rinsing with deionized (DI) water after each step. We fabricated the solar cells using an inverted structure. The bottom transport layer (ZnO), which acts as electron transport layer (ETL), was deposited using an automatic blade coater Zehntner ZAA 2300 with an aluminum applicator Zehntner ZUA 2000, in air conditions and at a constant speed of 5 mm s^−1^, with a drop volume of 50 μL, and temperature set at 40 °C. All active layer materials were deposited using a second blade coater equipment (same brand and model) that included custom‐made electronics to enable speed gradients, inside a nitrogen‐filled and dry glovebox, with a blade gap of 200 μm, and temperature set at different temperatures depending on the solvent: CPME at 60 °C, CB and *o*‐xylene at 80 °C, DCB at 105 °C. For mixtures we set the temperature to that corresponding to the solvent with highest boiling point. The drop volume used was 50 μL. For the processing of the thickness gradients the speed was configured as a linearly decelerating speed ramp, from 90 to 10 mm s^−1^. The top transport layer, which acts as hole transport layer (HTL), and electrode (MoOx/Ag) were thermally evaporated at a rate of 0.1 Å s^−1^ for the HTL and 1 Å s^−1^ for the metal electrode, respectively.

### Solar cell characterization

*J*–*V* characteristics were automatically obtained by using a Keithley source meter and an Arduino based multiplexer/switcher, which allows measuring 24 devices in less than 6 min. As a lighting source, a SAN‐EI Electric XES‐100S1 AAA solar simulator was used to ensure a homogeneous illumination in a 10 cm×10 cm area. The solar simulator was previously calibrated with a certified silicon solar cell (Oriel). EQE was measured with a homemade system that uses a supercontinuum light source (4 W, Fianium) coupled to a monochromator and normalized by the light power as measured by a silicon diode. We measured EQEs from 400 to 900 nm wavelength by focusing the laser on a spot of 50 mm in diameter.

### Thin film characterization

The film thickness was measured using a mechanical profilometer (Dektak 150, Bruker). The Raman scattering spectra and PL measurements performed in functional devices were acquired using a WITec alpha 300 RA+ confocal Raman setup, coupled to an Olympus objective with 10X magnification (NA 0.25). Two lasers centered at 488 and 633 nm were employed. The light was focused through the thick (1.1 mm), ITO‐covered glass substrates and the laser power reduced accordingly to avoid photodegradation and bleaching of the active layer (3–5 mW at 488 nm excitation). All raw data were collected using WITec Project FIVE piece of software and fitted with a custom‐made MATLAB software.^[52]^ Absorption spectra measurements were obtained from bibliography.^[16]^

## Conflict of interest

The authors declare no conflict of interest.

## Supporting information

As a service to our authors and readers, this journal provides supporting information supplied by the authors. Such materials are peer reviewed and may be re‐organized for online delivery, but are not copy‐edited or typeset. Technical support issues arising from supporting information (other than missing files) should be addressed to the authors.

Supporting Information

Supporting Information
