# Supplementary material for: High‐Throughput Screening of Blade‐Coated Polymer:Polymer Solar Cells: Solvent Determines Achievable Performance
Source: ChemSusChem. 2022 Jan 21;15(4):e202101888. doi: 10.1002/cssc.202101888 (PMC9305181; doi:10.1002/cssc.202101888)
Supplement: Supplementary file 1 — Supporting Information [file CSSC-15-0-s001.pdf]

ChemSusChem

Supporting Information

High-Throughput Screening of Blade-Coated Polymer: Polymer Solar Cells: Solvent Determines Achievable Performance

Albert Harillo-Baños, Qunping Fan, Sergi Riera-Galindo, Ergang Wang, Olle Inganäs,* and Mariano Campoy-Quiles*This publication is part of a collection of invited contributions focusing on “Advanced Organic Solar Cells”. Please visit to view all contributions.© 2021 The Authors. ChemSusChem published by Wiley-VCH GmbH. This is an open access article under the terms of the Creative Commons Attribution License, which permits use, distribution and reproduction in any medium, provided the original work is properly cited.

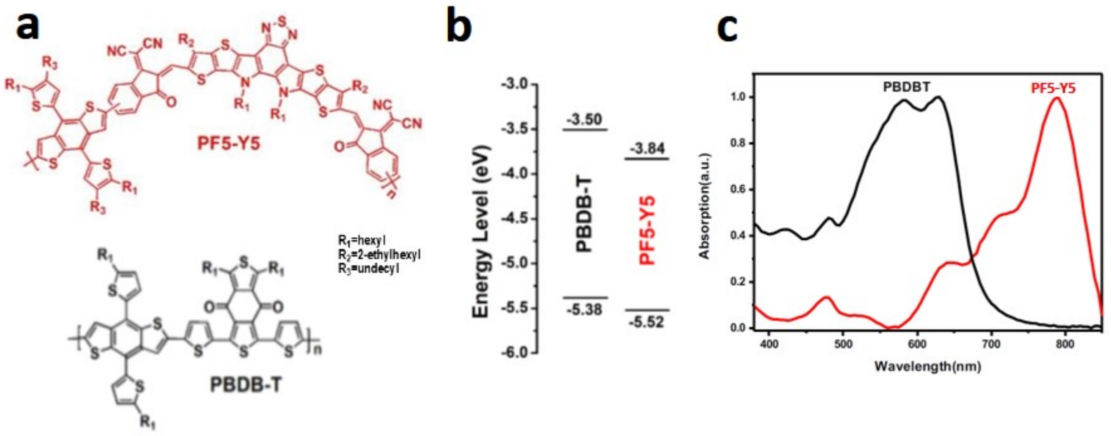

Figure S1. a) Chemical structures; b) Energy levels; and c) Normalized absorption of the two polymers [1]

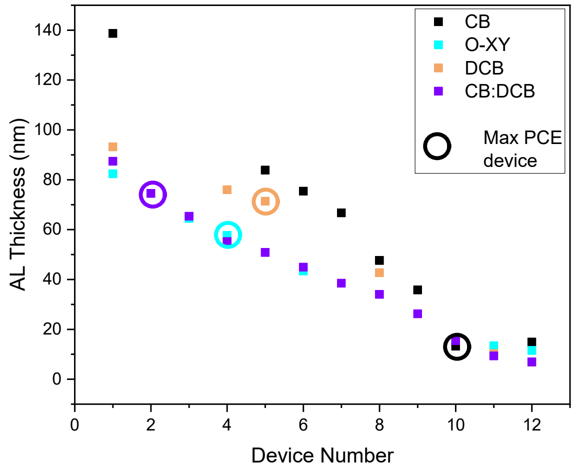

Figure S2: Thickness Gradient for each sample

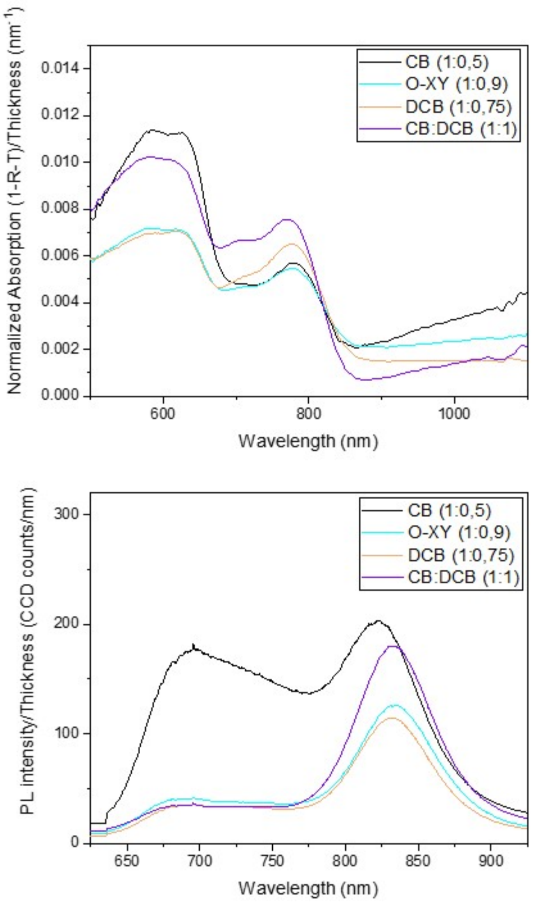

Figure S3: a) UV-Vis and b) PL measurements of the best device for each solvent. Between parenthesis is the D:A ratio of each device. In both cases, divided by the thickness of the Active Layer.

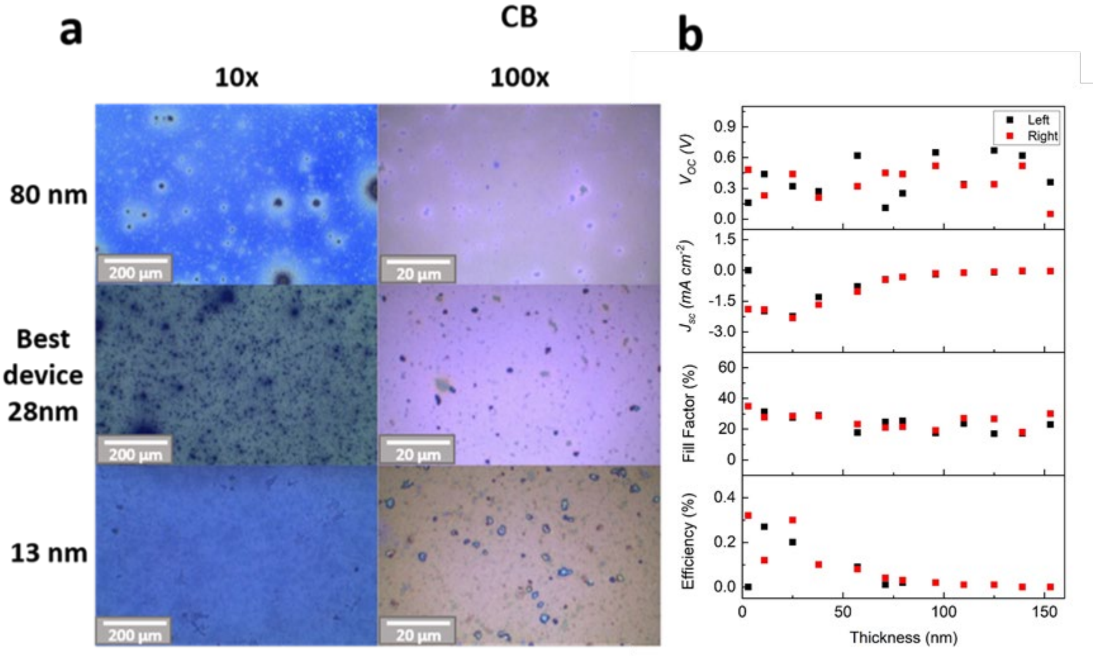

Figure S4: Optical microscopy images (10x and 100x) of the CB based sample

O-XY

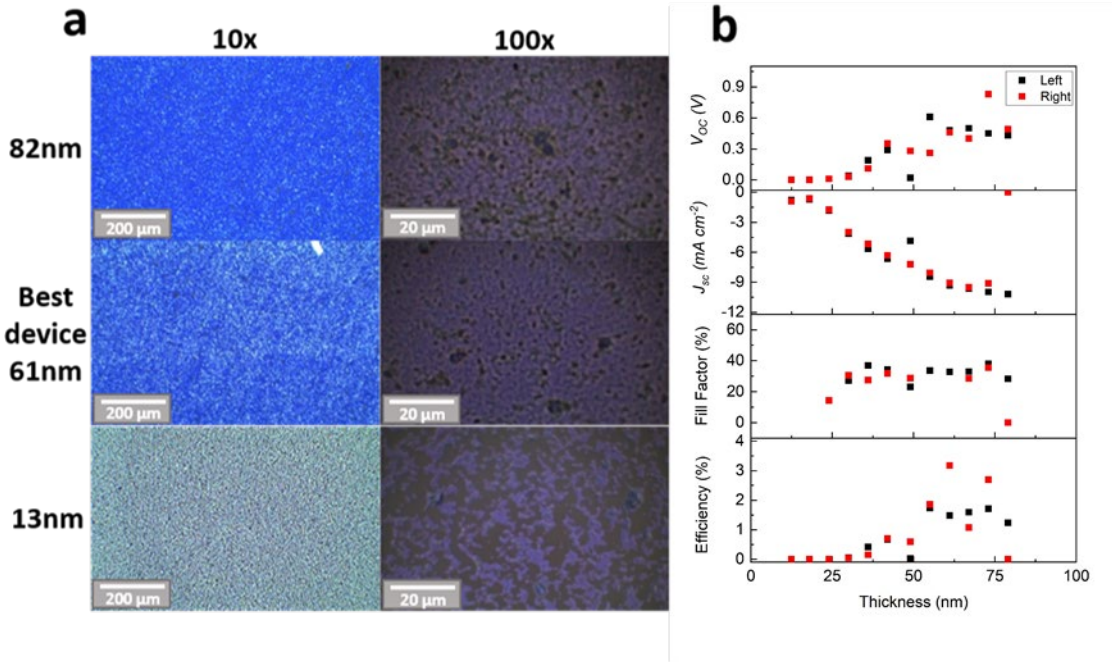

Figure S5: Optical microscopy images (10x and 100x) of the O-XY based sample

DCB

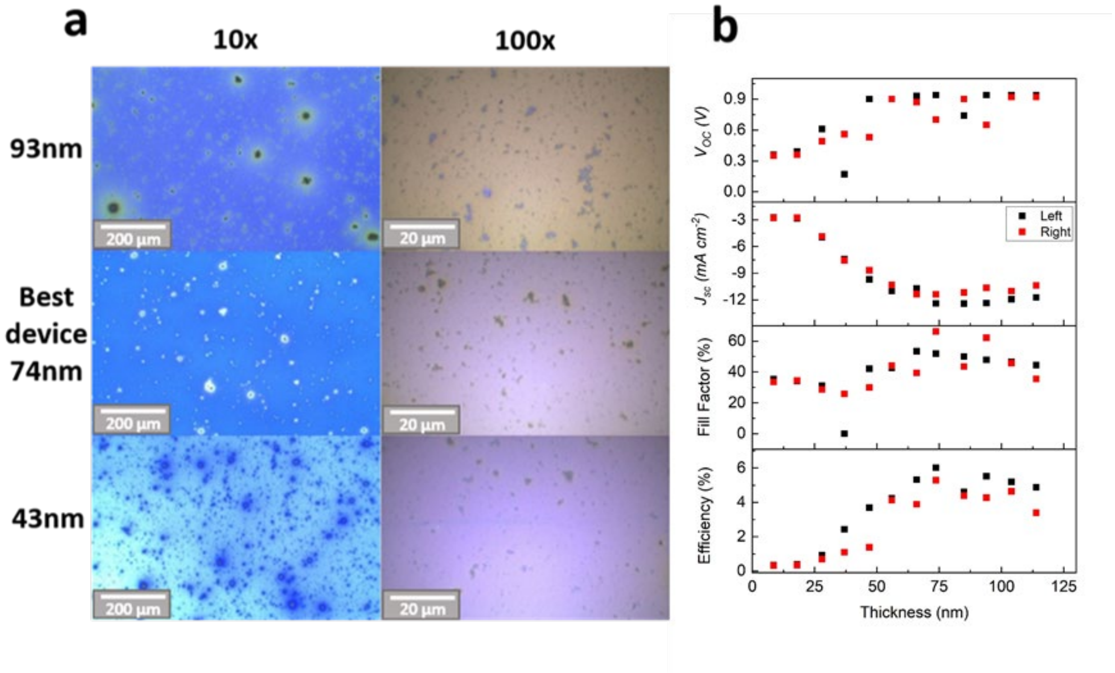

Figure S6: Optical microscopy images (10x and 100x) of the DCB based sample

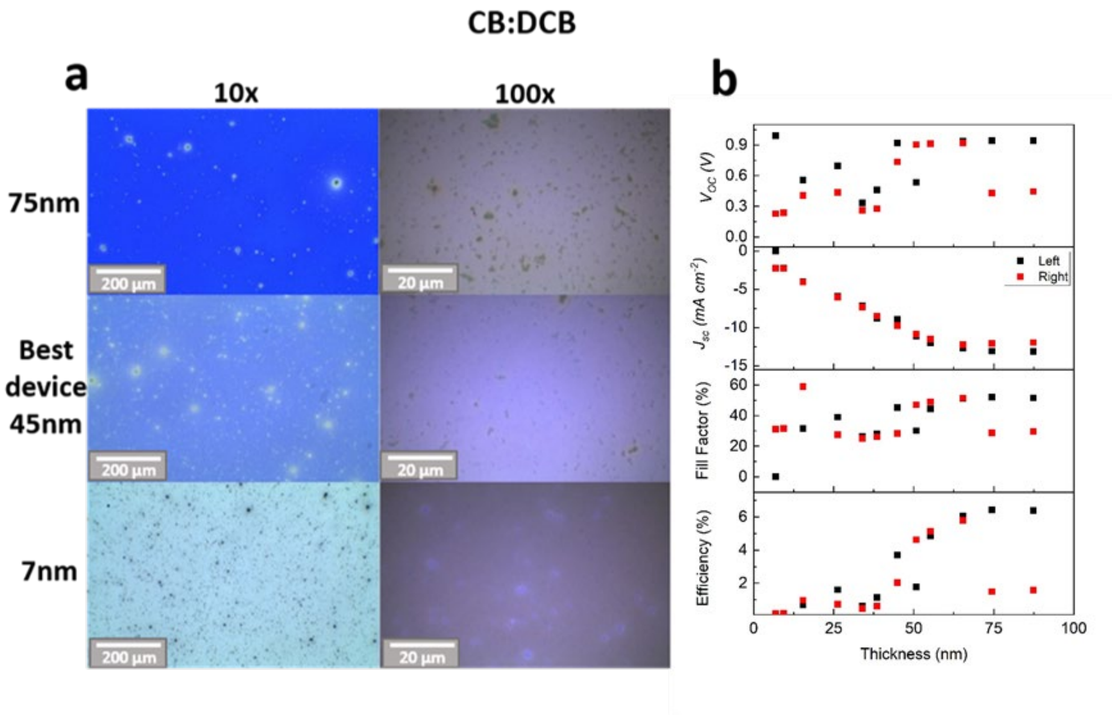

Figure S7: Optical microscopy images (10x and 100x) of the CB:DCB based sample

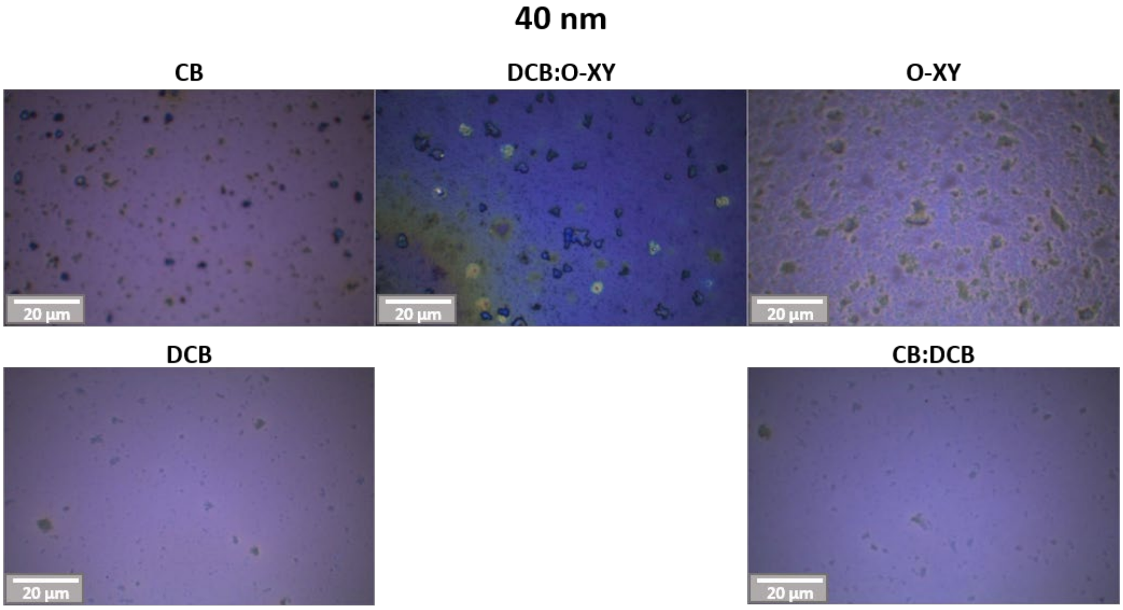

Figure S8: Optical microscopy images (100x) of different devices, with the same Active layer thickness (40nm).

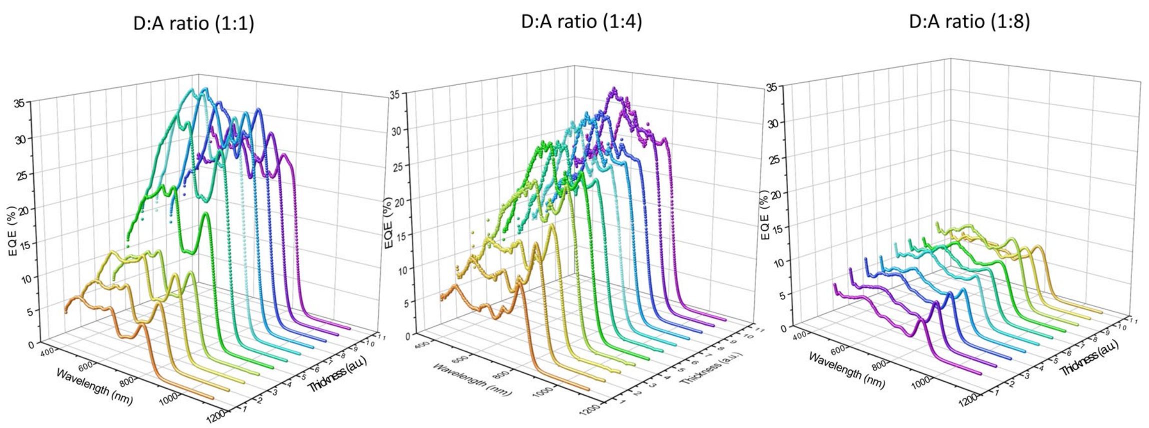

Figure S9: External Quantum Efficiency of PBDB-T:PF5-Y5 solar cells using DCB as a solvent at D:A ratio 1:1, 1:4 and 1:8 from left to right.

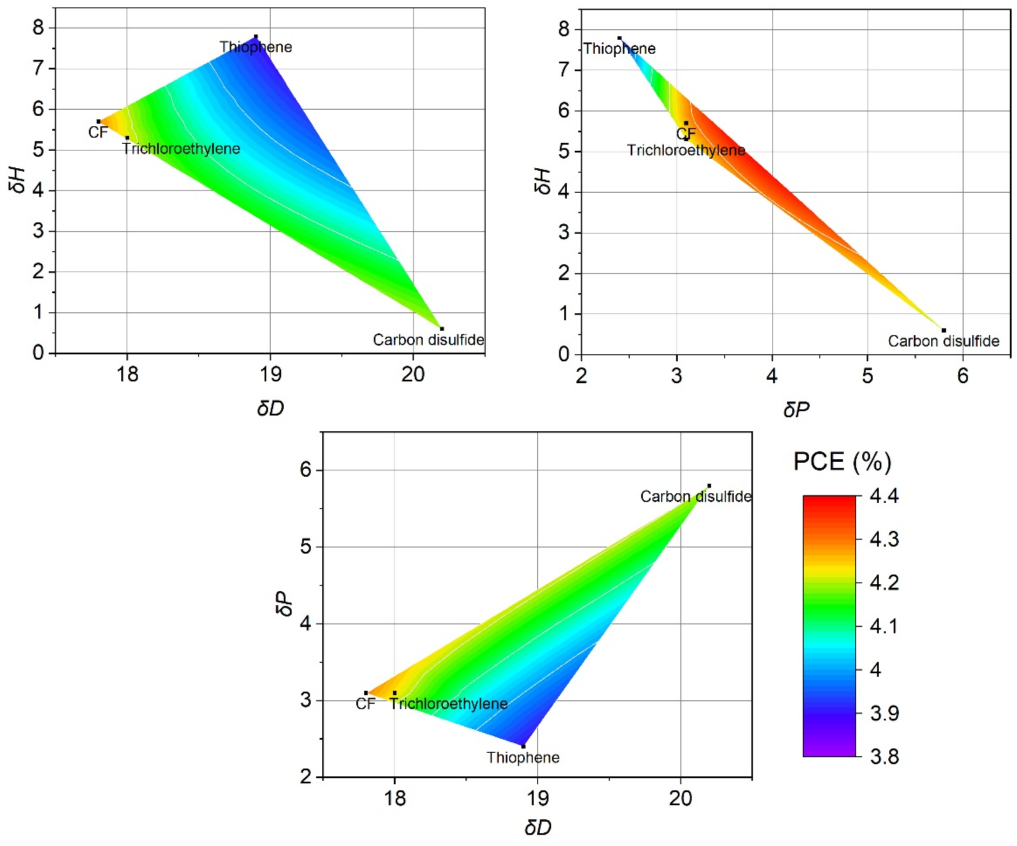

Figure S10. Performance landscape of DDP(TBFu)2:PC71BM solar cells represented on the Hansen solubility space, from bibliography [2]

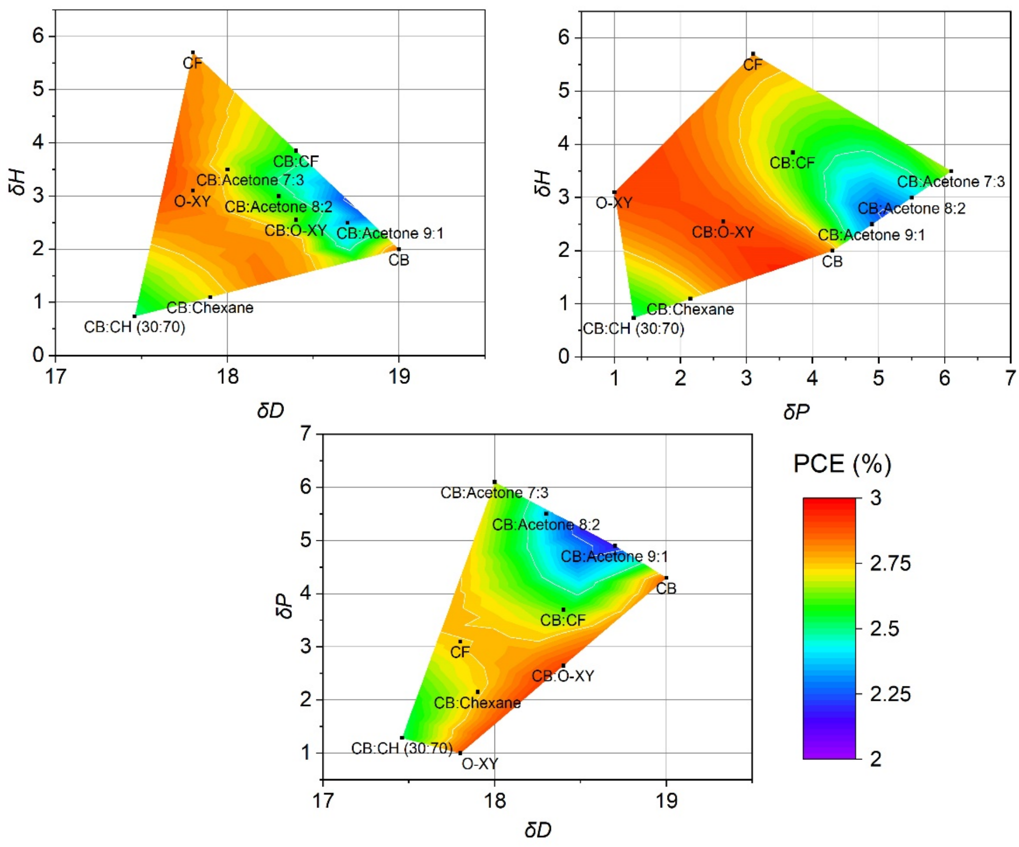

Figure S11. Performance landscape of P3HT:PC60BM solar cells represented on the Hansen solubility space, from bibliography [3]

[1] Q. Fan *et al.*, "Over 14% efficiency all-polymer solar cells enabled by a low bandgap polymer acceptor with low energy loss and efficient charge separation," *Energy Environ. Sci.*, vol. 13, no. 12, pp. 5017–5027, 2020.

[2] B. Walker *et al.*, "A systematic approach to solvent selection based on cohesive energy densities in a molecular bulk heterojunction system," *Adv. Energy Mater.*, vol. 1, no. 2, pp. 221–229, 2011.

[3] F. Machui, S. Abbott, D. Waller, M. Koppe, and C. J. Brabec, "Determination of solubility parameters for organic semiconductor formulations," *Macromol. Chem. Phys.*, vol. 212, no. 19, pp. 2159–2165, 2011.
